# A Further Analysis on Ti6Al4V Lattice Structures Manufactured by Selective Laser Melting

**DOI:** 10.1155/2019/3212594

**Published:** 2019-09-22

**Authors:** Saverio Maietta, Antonio Gloria, Giovanni Improta, Maria Richetta, Roberto De Santis, Massimo Martorelli

**Affiliations:** ^1^Department of Industrial Engineering, Fraunhofer JL IDEAS—University of Naples Federico II, P.le Tecchio 80, 80125 Naples, Italy; ^2^Institute of Polymers, Composites and Biomaterials—National Research Council of Italy, V.le J.F. Kennedy 54—Mostra d'Oltremare Pad. 20, 80125 Naples, Italy; ^3^Department of Public Health, University of Naples Federico II, via S. Pansini 5, 80131 Naples, Italy; ^4^Department of Industrial Engineering, University of Rome Tor Vergata, 00133 Rome, Italy

## Abstract

Mechanical and architectural features play an important role in designing biomedical devices. The use of materials (i.e., Ti6Al4V) with Young's modulus higher than those of natural tissues generally cause stress shielding effects, bone atrophy, and implant loosening. However, porous devices may be designed to reduce the implant stiffness and, consequently, to improve its stability by promoting tissue ingrowth. If porosity increases, mass transport properties, which are crucial for cell behavior and tissue ingrowth, increase, whereas mechanical properties decrease. As reported in the literature, it is always possible to tailor mass transport and mechanical properties of additively manufactured structures by varying the architectural features, as well as pore shape and size. Even though many studies have already been made on different porous structures with controlled morphology, the aim of current study was to provide only a further analysis on Ti6Al4V lattice structures manufactured by selective laser melting. Experimental and theoretical analyses also demonstrated the possibility to vary the architectural features, pore size, and geometry, without dramatically altering the mechanical performance of the structure.

## 1. Introduction

With regard to metal manufacturing, the relationship among process parameters, microstructure, and mechanical properties is fundamental in different areas (i.e., sintering, welding, casting, and plastic forming) and generally involves innovative and traditional fabrication methods [1–3].

Structural and mechanical properties play a crucial role in the design of biomedical devices with tailored performances which should satisfy the desired requirements.

As an example, functional devices for tissue reconstruction should resemble the structure and properties of the natural tissue.

In this context, metals like titanium (Ti) and its alloys have been widely employed. Titanium alloys are usually considered to make orthopedic and dental implants, due to their excellent biocompatibility, good corrosion resistance, and high strength [4–6]. Total hip replacements, interbody fusion devices for spinal applications, bone screws and nails, and parts of artificial heart valves are currently developed using titanium alloys [4, 7]. Stress shielding effects as well as bone atrophy and implant loosening are related to implants developed using a material with Young's modulus (i.e., Ti6Al4V, 114 GPa) which is much higher than that of the cortical bone (i.e., 10–30 GPa) [4, 8, 9].

Today, there is a great need to increase and to optimize implant life as a consequence of population ageing, diffusion of extreme sports, costs of implant replacement, and revision surgery [4].

Long-lasting implants may be clearly designed if materials with mechanical properties close to those of human tissues are employed [4].

An interesting strategy to reduce the implant stiffness would be the design of 3D porous devices with controlled geometry and architecture. A further advantage in designing porous implants is the possibility of tissue ingrowth, which should stabilize the implant [4, 9].

Several studies on bone reconstruction have already reported a correlation between newly formed tissue and pore size, as porous devices may offer surface and space favoring cell adhesion and bone ingrowth [4, 10]. Furthermore, pore interconnection is another important feature allowing for cell migration and *in vivo* blood vessel formation [4, 10, 11].

In general, many technologies have been considered to fabricate porous metal devices (i.e., direct laser metal sintering, selective laser melting, chemical vapor deposition, and space holder method) [4, 12–14] and also employed for titanium [4, 9, 15, 16].

On the contrary, it is well known how traditional techniques such as metal injection molding present some advantages resulting in a fast fabrication of high amount of complex parts with decreased production costs and in the possibility to modulate the properties through a suitable selection of powder size and/or sintering temperature [4]. Metal injection molding was successfully used to fabricate porous Ti6Al4V devices and mechanical, morphological, and biological properties were also evaluated [4].

Over the past years, the relationship between design and manufacturing has been explored in several fields. Differently from conventional fabrication methods, additive manufacturing technologies allow the direct production of customized and lightweight structures with improved properties [17–19].

The applicability of porous structures and lattices has been widely reported especially focusing on the development of orthopedic implants and 3D scaffolds for tissue engineering. As for the tissue regeneration approach, lattice structures should be biocompatible, biodegradable, and bioresorbable. They should be suitably optimized for cell attachment and growth, ensuring adequate mass transport and mechanical properties [18, 19].

In designing 3D lattice structures, a “unit cell” may be selected and a volume based on it may be built up, even if different methods are generally used to design the 3D periodic structures, also involving topology optimization and issues related to the implementation of the homogenization theory [19].

Ti6Al4V lattice components were already fabricated by selective laser melting and then analyzed in terms of surface roughness, microhardness, compressive properties, and dimensional accuracy [17]. In particular, microlattice structures were designed using the pillar textile unit cell as base and further lattice topologies which included reinforcing vertical bars were also considered [17].

Furthermore, a wide range of porosity (i.e., 60–95%) and unit cells with different sizes have been proposed for developing different kinds of Ti6Al4V components [17, 20], as well as implants (i.e., macropores in the range of 400–1000 *μ*m) which exhibited excellent osteointegration performance *in vivo* [21].

The aim of the current study was to provide a further theoretical-experimental analysis on Ti6Al4V lattice structures fabricated by SLM.

## 2. Materials and Methods

### 2.1. Design and Fabrication of 3D Lattice Structures

**Figure 1:**
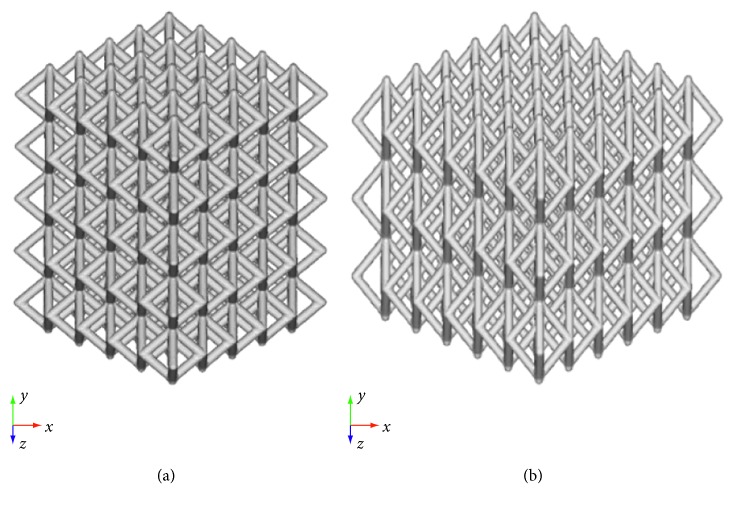
CAD models of lattice structures. (a) Model A. (b) Model B.

A computer-aided design- (CAD-) based approach was used to develop two kinds of morphologically controlled structures (Figure 1). Different cell units were designed, thus varying the pore size and using a strut diameter of 1 mm. SolidWorks® 2017 (Dassault Systemes, Paris, France) CAD system was used to carry the operations.

Two different kinds of additively manufactured structures were fabricated using an M2 Cusing SLM machine (Concept Laser, Lichtenfels, Germany) and Ti6Al4V powder (particle size: 45–105 *μ*m):

**Mode 1 l A.**

Lattice structure (25 mm × 25 mm × 25 mm) with 5 mm × 5 mm × 5 mm cell units.

**Mode 2 l B.**

Lattice structure (30 mm × 24 mm × 30 mm) with 5 mm × 8 mm × 5 mm cell units.

Lattice structures were fabricated with a laser beam of 170 W power, and scanning speed was set to 1250 mm/s [17, 22].

Further processing parameters were the spot size (100 *μ*m) and the oxygen content (<0.1%).

Additional heat treatment was performed at 650°C for 2 h in argon atmosphere, in order to avoid oxidation and to reduce internal stresses related to the high thermal gradients experienced during the fabrication process. Successively, the structures were subjected to sand blasting for partially removing molten particles [17].

### 2.2. Experimental and Theoretical Analyses

A stereomicroscope (Olympus SZX) and an image software were used to analyze the developed structures.

Experimental compression tests were carried out at a rate of 1 mm/min up to a load of 800 N, using an INSTRON 5566 testing system. Load-displacement curves were reported.

On the contrary, the IGES format was employed and the geometric models of the two morphologically controlled structures were imported into HyperMesh® (HyperWorks®—14.0, Altair Engineering Inc., Troy, Michigan, USA), which is a typical finite element (FE) preprocessor used for the management and the generation of complex models, starting with the import of CAD geometry to exporting ready-to-run solver file.

FE analysis was performed on the developed models in order to simulate the experimental tests. Young's modulus and Poisson's ratio for Ti6Al4V are reported in Table 1.

The mesh was generated, and 3D solid CTETRA elements with four grid points were employed. Appropriate mesh size and refinement techniques were also used.  Table 2 reports some technical features (i.e., total number of grids-structural, elements excluding contact, node-to-node surface contact elements, and degrees of freedom) for models A and B.

In addition, “freeze” type was used as contact conditions between the model and the compression plates. Constraints were applied for nodal displacements in all the directions. The external surface of the lower plate was constrained, whereas a compression load of 800 N acted on the external surface of the upper plate.

Linear static analyses were performed taking into account a nonfailure condition. Displacements, maximum principal stress, and von Mises stress distributions were evaluated.

## 3. Results and Discussion

In the biomedical field, it has been reported that the great mismatch between the stiffness of metal devices and surrounding tissues causes stress concentration and stress shielding effects, thus frequently leading to the implant loosening [4]. Anyway, it is also worth underlining that the implant stiffness, which determines stress distributions, depends not only on Young's modulus (an intrinsic mechanical property of the material) but also on the shape and size [4, 24, 25].

The properties of a device may be varied focusing on the material-shape combination and, hence, an appropriate combination of CAD-based approach and material selection. However, the important role of CAD and experimental and theoretical analyses has been frequently discussed for different kinds of biomedical applications [24–28].

To overcome the drawbacks (i.e., stress shielding effects, bone atrophy, and implant loosening) related to the use of materials (i.e., Ti6Al4V) with Young's modulus higher than those of natural tissues, the design of porous devices may be considered for the reduction of the implant stiffness and, consequently, for the improvement of its stability by promoting tissue ingrowth [4].

Porosity and architecture may be taken into account to tailor mechanical and functional features of biomedical device. As an example, in the case of devices for tissue reconstruction and regeneration, if porosity increases, mass transport properties, which are crucial for cell behavior and tissue ingrowth, increase, whereas mechanical properties decrease [18].

Porous devices fabricated by additive manufacturing have the potential to be the next step in the development of up-to-date biomedical implants for several applications (i.e., bone reconstruction, dental implants, and interbody fusion devices).

Many attempts have been made to define architectural features, pore shape, and size, according to the mechanical and mass transport properties required for a specific application, also involving topology optimization, implementation of the homogenization theory, and well-defined algorithms and procedures. In the literature, many works report the potential to tailor mass transport and mechanical properties of additively manufactured structures by varying geometry, architectural features, as well as pore shape and size [18, 19].

The future looks bright for the additive manufacturing as it offers the possibility to design advanced and patient-specific implants in modern surgery. Accordingly, an individualized 3D printed Ti6Al4V cage for spinal cervical fusion was also proposed and developed integrating different approaches such as virtual reality simulation, CAD planning, and additive manufacturing [29]. In this case, a porous macro- and microcellular trabecular architecture was designed to improve osseointegration, and the porous cage was manufactured using SLM. Preliminary surgical implantations evidenced interesting results especially in terms of primary stability [29].

Furthermore, researchers already focused the attention on the design of Ti6Al4V implants by varying porosity in a wide range (i.e., up to 95%) and unit cells with different sizes (i.e., pores in the range of 400–1000 *μ*m), which provided excellent osteointegration performance *in vivo* [21].

In this context, the present study would represent only a further step towards the analysis of Ti6Al4V lattice structures designed using different architectural features and cell units, trying to improve the knowledge of the structure-property relationship through experimental and theoretical analyses.

**Figure 2:**
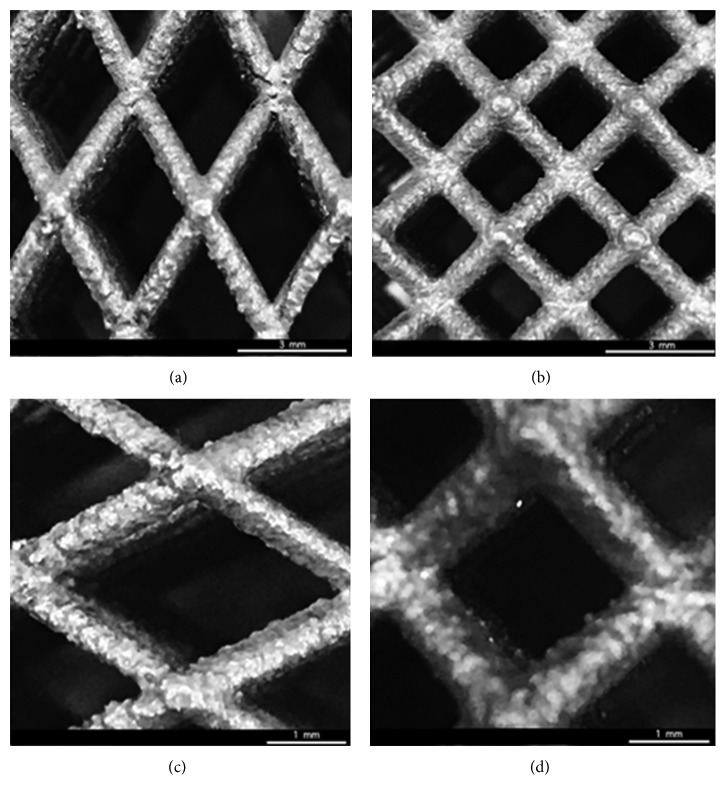
Results from morphological analysis performed on models B (a, c) and A (b, d) of Ti6Al4V lattice structures, using a stereomicroscope (Olympus SZX) and an image software.

Morphological analysis on the manufactured models allowed to study the structural features and the internal architecture of the devices and to confirm the consistency between theoretical values defined by the CAD-based approach and the experimental ones for the strut diameter and the cell unit sizes (Figure 2).

**Figure 3:**
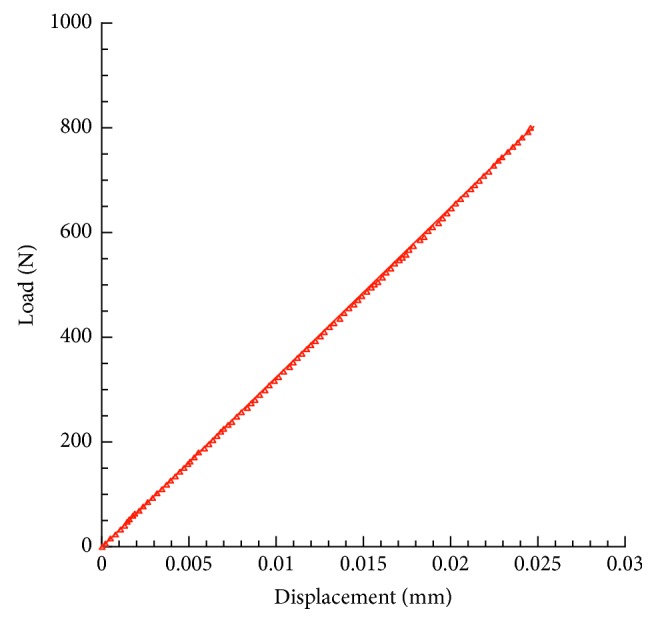
Results from experimental compression tests. Typical load-displacement curve obtained for model A of Ti6Al4V lattice.

**Figure 4:**
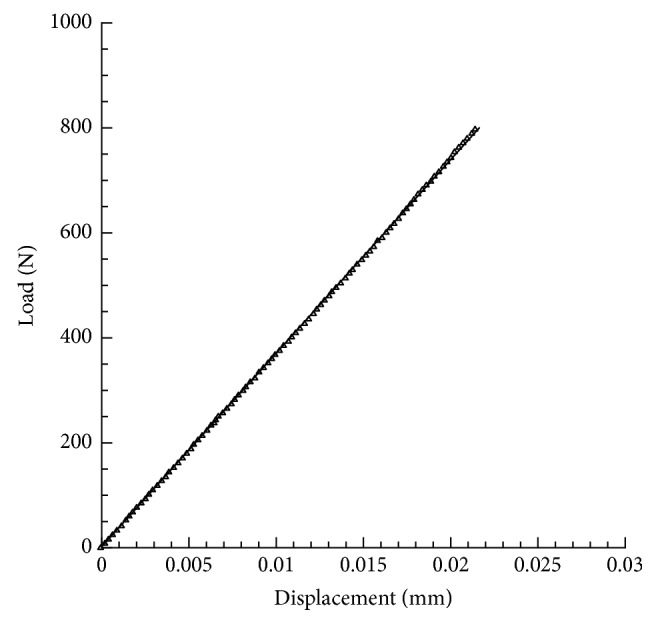
Results from experimental compression tests. Typical load-displacement curve obtained for model B of Ti6Al4V lattice.

Results from experimental compression tests provided load-displacement curves which were linear up to a load level of 800 N (Figures 3 and 4).

In terms of mean value ± standard deviation, displacements of 0.023 ± 0.002 mm and 0.021 ± 0.002 mm were experimentally observed for models A and B, respectively.

On the contrary, FE analysis, which was carried out to simulate the experimental tests, allowed to obtain displacements, maximum principal stress, and von Mises stress distributions.

**Figure 5:**
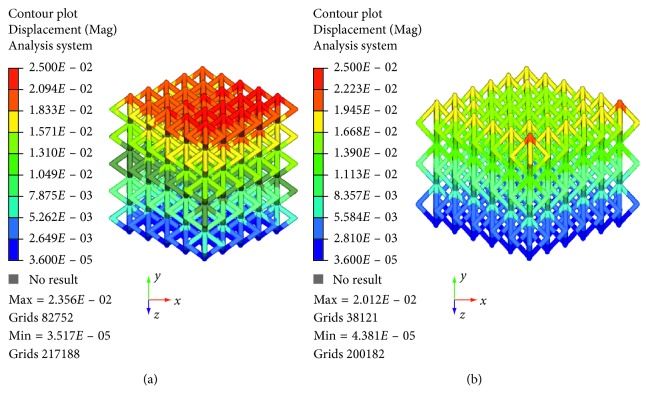
Results from FE analysis: displacement (mm) contour plot for model A (a) and model B (b) of Ti6Al4V lattice structures.

Specifically, Figure 5 reports the displacement contour plot for models A and B of Ti6Al4V lattice structures.

As expected on the basis of the results from the experimental tests, models A and B of Ti6Al4V lattice structures provided a maximum displacement value of about 0.024 mm and 0.020 mm, respectively. Thus, the results obtained from theoretical analyses were in agreement with the experimental ones.

**Figure 6:**
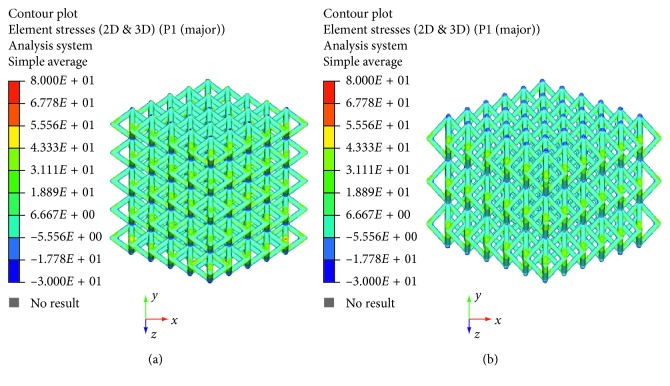
Results from FE analysis: maximum principal stress (MPa) distribution for model A (a) and model B (b) of Ti6Al4V lattice structures.

**Figure 7:**
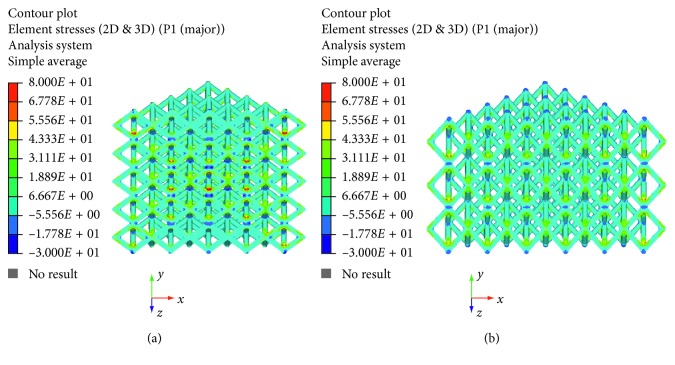
Results from FE analysis: maximum principal stress (MPa) distribution for model A (a) and model B (b) of Ti6Al4V lattice structures.

**Figure 8:**
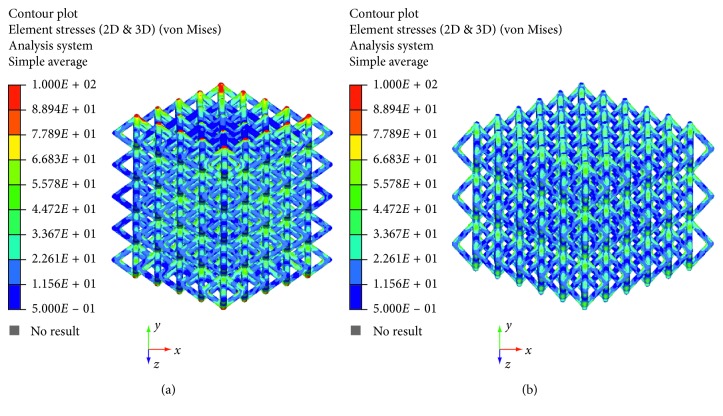
Results from FE analysis: von Mises stress (MPa) distribution for model A (a) and model B (b) of Ti6Al4V lattice structures.

**Figure 9:**
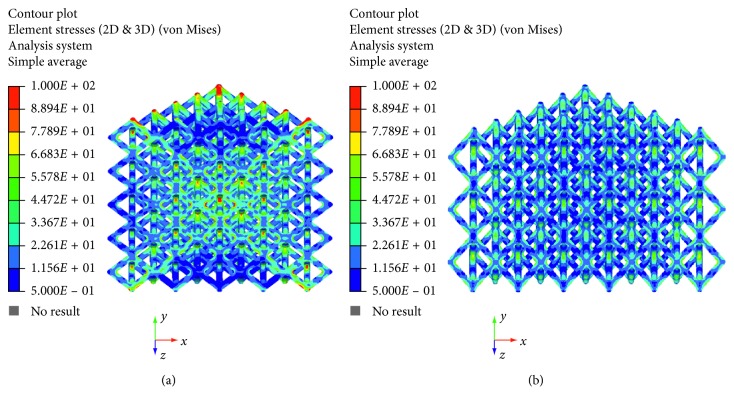
Results from FE analysis: von Mises stress (MPa) distribution for model A (a) and model B (b) of Ti6Al4V lattice structures.

The maximum principal stress and von Mises stress distributions were also evaluated and reported in Figures 6–9.

The linear load-displacement (and, hence, stress-strain) curves together with the maximum principal stress and von Mises stress distributions showed that plastic deformation was not present also at local levels.

Figures 6–9 evidence some differences in terms of stress distribution between the two proposed models according to the obtained values for the displacement (Figure 5), as a direct consequence of geometry, architectural features, and unit cell.

**Figure 10:**
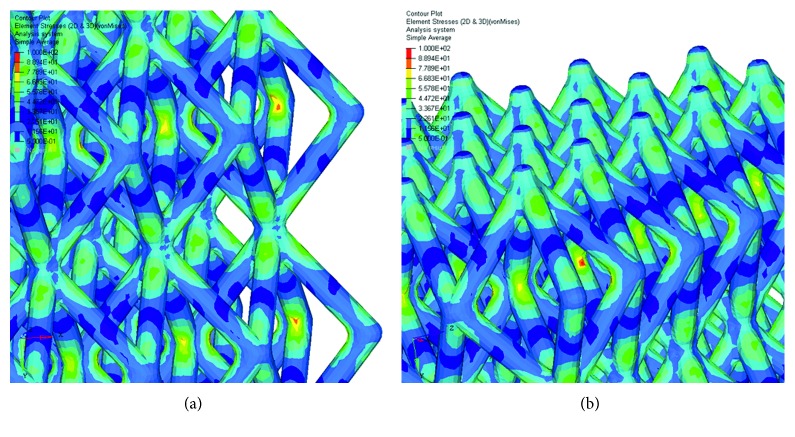
Results from FE analysis: von Mises stress (MPa) distribution for model B of Ti6Al4V lattice structures. Further section views (a) and (b). The color scale was chosen to allow comparisons.

Even though in terms of stress distributions, some differences were found (Figures 6–9), as a greater number of regions with high local stress gradients were observed for model A in comparison to model B, and similar values of maximum stress were achieved for both models (i.e., von Mises stress of about 94–100 MPa) (Figures 6–10). In particular, although Figures 6–9 would evidence higher values of stress for model A in comparison to model B, Figure 10 reports an example of further section views for model B which better shows small local areas where the stress reached values similar to those found for model A. Thus, under the same loading condition, differences in geometrical and architectural features led to different stress distributions, however providing similar values of maximum stress, even if in model B they were achieved in smaller local areas.

Taking into account a methodology already reported for 3D porous structures with controlled architectural features [18], the “apparent” stress (*σ*) and strain (*ε*) can be also evaluated (*σ* = *F*/*A*_0_, *ε* = Δ*H*/*H*_0_), when the values of the force (*F*) measured by the load cell, the apparent initial cross-sectional area (*A*_0_), the initial height (*H*_0_), and the height variation (Δ*H*) of the structure are known.

Accordingly, starting from the above-reported considerations together with the obtained results, a compressive modulus of 1.4 ± 0.1 GPa and 1.1 ± 0.1 GPa can be determined for model A and model B, respectively.

However, it is worth noting that, even though in the case of porous structures, the compressive modulus does not represent the elastic modulus, which is an intrinsic property of the material, and it clearly provides information on the stiffness of the designed structures.

Generally, structures for biomedical applications consist of materials like titanium and its alloys or other metals showing plastic deformation and yielding before fracture.

Devices must be designed so that they are loaded within the elastic limit and, clearly, do not yield.

Furthermore, it is well known how the heat treatment affects the behavior of the structures [17, 22].

For this reason, a static linear analysis was first performed using a nonfailure condition, and the present work would be a further study towards a future research with the aim of developing a complex model to describe the mechanical behavior and failure mechanisms of lattice structures manufactured by SLM, starting from the structure-property relationship.

For this reason, some potential limitations of the present research were as follows: (i) the linear static analysis carried out considering a nonfailure condition and the consequent lack of information on the strength and ductility of the manufactured structures, both at the theoretical and experimental levels and (ii) no evaluations of the effect of different heat treatments on the mechanical behavior.

## 4. Conclusions

Despite the limitations of the current analysis, the following conclusions were drawn:

- Structural features and internal architecture were visualized, and the consistency between theoretical values defined by the CAD-based approach and the experimental ones for the strut diameter and the cell unit sizes was verified.
- The theoretical analyses performed on the two models were able to predict the experimental values for the displacement (0.023 ± 0.002 mm and 0.021 ± 0.002 mm) obtained from compression tests, also confirming the important role of CAD-FE modelling in the study of devices which could be predesigned to match the mechanical properties of natural tissues.
- The possibility to tailor the architectural features, pore shape and size, and, eventually, mass transport properties, without dramatically altering the mechanical performance of the porous device, was confirmed.

## Figures and Tables

**Table 1:** Young's modulus and Poisson's ratio employed for Ti6Al4V [4, 23].

Young's modulus (GPa)	Poisson's ratio
114	0.33

**Table 2:** Total number of grids (structural), elements excluding contact, node-to-node surface contact elements, and degrees of freedom.

Model	Total number of grids (structural)	Total number of elements excluding contact	Total number of node-to-surface contact elements	Total number of degrees of freedom
A	445,484	1,903,264	1,346	1,328,773
B	581,235	2,171,639	2,952	1,726,830

## Data Availability

All data generated or analyzed during this study are included in this article.
